# Mutational screening of Indian families with hereditary congenital cataract

**Published:** 2013-05-29

**Authors:** Surya Prakash Goud Ponnam, Kekkunaya Ramesha, Jyoti Matalia, Sushma Tejwani, Balasubramanya Ramamurthy, Chitra Kannabiran

**Affiliations:** 1 Kallam Anji Reddy Molecular Genetics Laboratory, L V Prasad Eye Institute, KAR Campus, Banjara Hills Road No.2, Hyderabad, Andhra Pradesh, India; 2 Jasti V Ramanamma Children’s Eye Care Centre, L V Prasad Eye Institute, KAR Campus, Banjara Hills Road No.2, Hyderabad, Andhra Pradesh, India; 3 Department of Molecular Biology and Biotechnology, Tezpur University, Napaam, Sonitpur, Assam, India; 4 Narayana Nethralaya, Narayana Health City, Bangalore, India; 5 Aravind Eye Hospital, Mehdipatnam, Hyderabad, India

## Abstract

**Purpose:**

To screen for pathogenic mutations in ten candidate genes in Indian families diagnosed with autosomal recessive and autosomal dominant cataracts.

**Methods:**

Families with two or more affected individuals with bilateral familial congenital/developmental cataract were ophthalmically evaluated, and blood samples were obtained. Genomic DNA extracted from the blood leukocytes was screened with PCR amplification of the exons and the flanking intronic regions of various genes selected for analysis. The amplified products were subjected to single strand conformation polymorphism (SSCP) analysis. The variants in SSCP analysis were subjected to bidirectional sequencing by automated methods.

**Results:**

We identified four novel sequence changes that cosegregated with the disease phenotype in each family and were absent in at least 50 ethnically matched unrelated normal controls. These changes include a homozygous missense change of c.649G>A (Val196Met) in *GJA8*/connexin 50 (Cx50) in a family with autosomal recessive cataract, two heterozygous missense changes, c.658C>T (Pro199Ser) in *GJA8*/Cx50 and c.589C>T (Pro197Ser) in *GJA3*/connexin 46 (Cx46) in two separate families with autosomal dominant cataract, and a silent change ( c.84G>A/p.Val28Val, predicted to result in the creation of a new potential branch point) in *GJA8* one family with an autosomal dominant inheritance of cataract. Of the four novel mutations identified, three mutations, Val196Met (*GJA8*), Pro199Ser (*GJA8*), and Pro197Ser (*GJA3*), are predicted to be in the second extracellular domain of the respective connexin proteins.

**Conclusions:**

Our report extends the mutation spectrum of connexin genes *GJA8* and *GJA3* and confirms that connexin genes are among the most frequently mutated genes in hereditary cataracts. Our results suggest that connexin gene (*GJA8* and *GJA3*) mutations occur in approximately 10% (4/40 families) of families with congenital hereditary cataracts in a population from southern India.

## Introduction

Cataract is defined as any opacity of the lens that impairs vision. As these opacities can cause blurring of vision during development by interfering with the sharp focus of light on the retina, they may result in failure to establish appropriate visual cortical synaptic connections with the retina, and result in permanent visual loss. Hereditary cataracts are clinically and genetically heterogeneous, often presenting as congenital or developmental cataracts that arise at birth or during the first few decades of life. There is a wide variation in the prevalence of childhood cataracts depending on the ascertainment methods [1]. Although the prevalence of congenital cataracts is 1~6 in 10,000, the incidence is 2.2 to 2.49 per 10,000 live births in Western countries [2-5]. Congenital cataract is one of the major causes of childhood blindness in India [6,7]. Approximately 25% of non-syndromic cataracts are inherited [8]. Congenital hereditary non-syndromic cataracts can be autosomal dominant (AD), autosomal recessive, or X-linked with the autosomal dominant form the most prevalent mode of inheritance [9]. Alternatively, they can also be grouped into three major classes, based on the functions of known underlying genes, those that code for crystallins, membrane/cytoskeleton proteins, and transcription factors. At least 35 independent loci, including more than 20 known genes, have been identified for non-syndromic cataract (Cat-Map) [10,11]. Mutations in crystallin genes account for the majority of missense mutations (nearly 50%) followed by mutations in the genes for cytoskeletal or membrane proteins (nearly 35%) [10]. Although the *GJA8* and *GJA3* mutations together account for 20% of the reported total non-syndromic familial cataracts worldwide [10], Devi et al. have reported that mutations in *GJA8* cause 3.3% of congenital cataracts in patients from southern India [12].

We selected ten candidate genes that are relatively frequently reported to have mutations in hereditary congenital cataract to date to identify mutations in families with congenital hereditary cataracts in the present study. The ten genes screened included six genes coding for crystallin proteins (*CRYAA*, *CRYBA3/A1*, *CRYBB2*, *CRYGB*, *CRYGC*, *CRYGD*), two genes for lens gap junction proteins (*GJA3* and *GJA8* for connexin 46 and 50 proteins, respectively), one gene for heat shock transcription factor (*HSF4*), and one gene for intrinsic membrane protein *LIM2*.

## Methods

### Clinical evaluation and DNA samples

The study protocol was approved by the Institutional Review Board of L. V. Prasad Eye Institute, Hyderabad and adhered to the guidelines of the Declaration of Helsinki. Probands and family members were recruited at the Jasti V Ramanamma Children’s Eye Care Centre, L V Prasad Eye Institute, Hyderabad, for the study. Forty families composed of 184 individuals (100 affected and 84 unaffected, 90 males and 94 females) with autosomal recessive and autosomal dominant forms of congenital hereditary cataracts were included. The ages of affected probands and their relatives at the time of presentation at our institution ranged from 0-45 years. The subjects had no systemic diseases or developmental disorders and were otherwise healthy at the time of recruitment. Eligible probands with hereditary cataract either with a diagnosis of bilateral congenital or developmental cataract with two or more affected individuals in the family were recruited for the study. All first-degree relatives of the proband and, if there was history of disease in other relatives, all unaffected and affected members of those branches of the family that had the disease, were recruited after receiving prior written informed consent from the study subjects or their guardians (in the case of minor subjects). Diagnosis of hereditary cataract was based on the presence of a bilateral familial lenticular opacity of any size of congenital or developmental type (age of onset, 0-16 years) as evaluated independently by two examiners. Patients with a history of trauma, or having unilateral (nonfamilial) cataract, co-existing ocular disease, mental retardation, microcephaly, cerebral palsy, systemic syndromes, or a maternal history of intrauterine infections or antenatal steroid use were excluded [13,14].

### Mutation analysis

Peripheral blood was collected by venipuncture in EDTA-coated vaccutainers, and stored at -20 °C until further use. Isolation of genomic DNA from peripheral blood leukocytes was performed by standard methods involving phenol-chloroform extraction. The DNA was quantitated spectrophotometrically (UV-1601, Shimadzu Corporation, Tokyo, Japan). Genomic or cDNA sequences of the genes were obtained from the Ensembl database or Vega database. Ensembl/Vega transcript IDs of the genes screened in this study are listed. For nomenclature of sequence changes, numbering of residues referred to the cDNA sequence and started with the first base of the initiation codon (ATG). Suitable primers were designed using the Primer3 software to generate amplified products of 350 bp or less for single strand conformation polymorphism (SSCP) analysis, such that they span the exons and the flanking intronic regions of the candidate genes. Primers were commercially obtained. The PCR products obtained were subjected to SSCP analysis. Primers were designed to obtain fragments of <300 bp in length for single-strand conformation polymorphism (SSCP) analysis. PCR products were mixed with two volumes of formamide, denatured by heating at 90 °C, snap-chilled and loaded on to 8% non-denaturing polyacrylamide gels with 5% glycerol. All samples were subjected to electrophoresis at 4 °C and at room temperature. Gels were fixed and subsequently stained with silver nitrate, and DNA visualised under visible light. The variants detected with SSCP analysis were subjected to bidirectional sequencing by automated methods. The observed changes were evaluated for segregation within the available family members, and each novel sequence change detected was tested for presence or absence in at least 75 ethnically matched unrelated normal controls with either SSCP or restriction fraction length polymorphism (RFLP) analysis if applicable, or direct sequencing. The restriction enzyme digestions were according to the manufacturers’ protocols. The digested products were then analyzed by subjecting them to 10% acrylamide gel electrophoresis in 1X Tris-borate-EDTA (TBE), and the DNA was visualized by staining with ethidium bromide.

### Analysis of sequence variations

Online tools including Sorting Intolerant From Tolerant (SIFT) [15] and Polymorphism Phenotyping v2 (PolyPhen-2) were used to predict the possible effects of the observed missense mutations on the proteins [16]. The Human Splicing Finder (HSF) was used to assess the potential impact of the silent change on messenger RNA (mRNA) splicing [17]. SIFT is used to predict the effect of sequence changes on the protein’s function, based on homology search and the physical properties of amino acids (SIFT). SIFT scores range from 0 to 1, and scores below 0.05 suggest that the amino acid change is not tolerated. PolyPhen-2 is a tool that predicts the possible impact of an amino acid substitution on the structure and function of a human protein using straightforward physical and comparative considerations (PolyPhen-2). PolyPhen-2 scores >0.85 are interpreted as probably damaging and scores 0.15–0.85 as possibly damaging. The HSF tool helps in predicting the effects of mutations of splicing signals or in identifying splice motifs, if any, in the sequences analyzed.

## Results

Forty families composed of 184 individuals (100 affected and 84 unaffected) with autosomal recessive and autosomal dominant forms of congenital hereditary cataracts were recruited in the study. Based on pedigree information, family history, and affection status of individuals recruited, 30 families had autosomal dominant cataracts, and ten families had autosomal recessive cataracts. After analyzing the ten selected candidate genes, we identified four novel sequence changes. In *GJA8*/Cx50, a homozygous missense change of c.649G>A (Val196Met) was found in one family with autosomal recessive cataract and one heterozygous missense change, c.658C>T (Pro199Ser), in one family with dominant cataract. Two sequence changes were identified in *GJA3*/Cx46: a missense change c.589C>T (Pro197Ser) and a silent change c.84G>A (Val28Val), respectively, in two separate families with autosomal dominant cataract.

**Figure 1:**
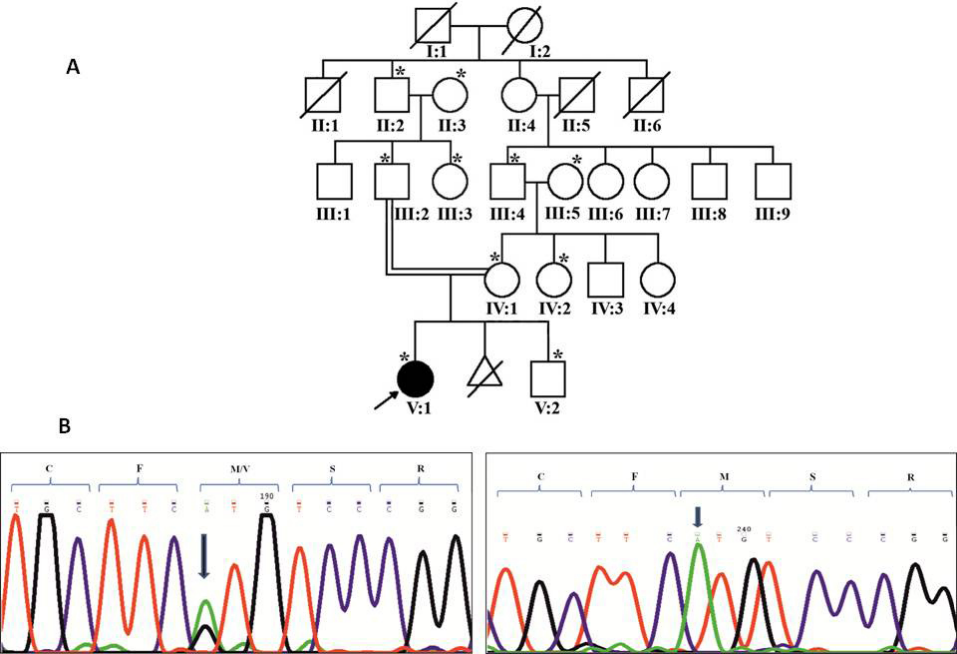
Family 32 with substitution Val196Met in *GJA8*. **A**: The pedigree of family 32 with autosomal recessive cataract is shown. Squares and circles represent men and women, respectively. Filled and clear symbols denote affected and unaffected individuals, respectively. Symbols marked with asterisks represent individuals who were genetically tested. **B**: The sequence electropherograms of the individuals screened in Family 32 are shown. The sequence for GJA8 shows homozygous substitution of G>A (c.649G>A) in the proband (right panel) whereas the unaffected mother was heterozygous for the same (left panel). The arrow shows the sequence change with codons marked with brackets.

In family 32 (Figure 1A) with autosomal recessive cataract, the proband (V:1) was diagnosed with congenital cataract and presented to the clinic at the age of 20 months. On genetic screening, a homozygous substitution of c.649G>A in exon 2 of *GJA8*/Cx50 resulting in Val196Met was observed (shown in Figure 1B). The proband’s parents (III:2 and IV:1), a sibling (V:2), and other relatives, including the proband’s maternal aunt, maternal grandfather, and paternal grandfather (IV:4, III:4, and II:2 respectively, in Figure 1A), were heterozygous for this change (Figure 1B). Individuals III:3, III:5, and II:3 (Figure 1A) had two copies of the normal allele. The proband’s mother had a few dot-like opacities in the lens and had an unaided visual acuity of 6/6 and N6 in both eyes at the age of 25 years at first presentation, suggesting that these opacities were not significant as they did not interfere with vision. The remaining members of this family showed normal lenses upon evaluation. Screening of 75 ethically matched normal controls with SSCP showed this change was absent.

**Figure 2:**
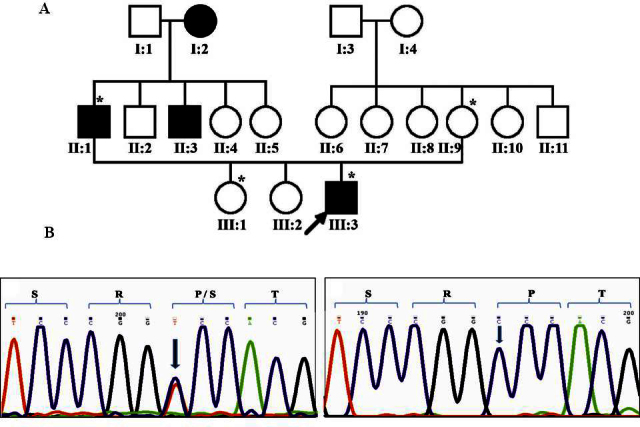
Family 21 with substitution Pro199Ser in *GJA8*. **A**: The pedigree of family 21 with autosomal dominant cataract is shown. Symbols marked with asterisks represent individuals who were genetically tested. **B**: The sequence for *GJA8* shows the heterozygous substitution of CCC>TCC (Pro->Ser; c658C>T) in the proband (left panel), whereas the unaffected mother had two normal alleles (right panel). The sequence change is indicated with an arrow.

In family 21 (Figure 2A), with autosomal dominant cataract, a heterozygous substitution of c.658C>T in exon 2 of *GJA8*/Cx50 (Figure 2B), resulting in missense change Pro199Ser, was observed in the proband (III:3) and his father (II:1) whereas the proband’s elder sister and his mother (III:1 and II:9, respectively) had two copies of the normal allele (Figure 2B). The sequence change resulted in creation of a recognition site for the enzymes HaeIII and *BsuR1*. The proband presented at 7 months of age and reportedly had cataracts since 3 months of age. His sibling aged 7 years at presentation was normal. The proband’s father (34 years at presentation) reportedly had been affected since birth and had previously undergone cataract surgery in both eyes. The proband’s mother was normal at age 25 years. Seventy-five ethnically matched unrelated normal controls were tested for this change with PCR-RFLP analysis with the *BSuR1* enzyme, and it was found to be absent.

**Figure 3:**
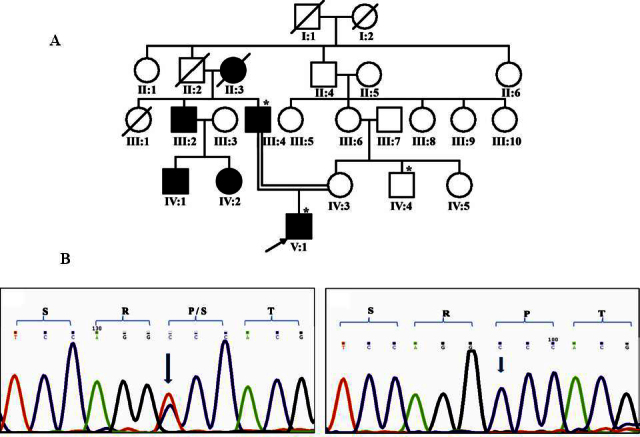
Family 41 with Pro197Ser in *GJA3*. **A**: The pedigree of family 41 with autosomal dominant cataract is shown. Symbols marked with asterisks represent individuals who were genetically tested. **B**: Representative electropherograms of the individuals screened in family 41 with heterozygous substitution of c.589C>T in the affected individual (left panel) and a normal sequence (right panel) are shown.

In family 41 (Figure 3A), a heterozygous substitution c.589C>T in exon 2 of *GJA3*/connexin 46 resulting in Pro197Ser was observed in both affected individuals (III:4 and V:1), whereas an unaffected individual who was the maternal uncle of the proband (IV:4) had two normal alleles (Figure 3B). The observed change is predicted to be in the second extracellular domain of the *GJA3*/Cx46 protein. This change resulted in creation of a recognition site for the AvaI*I* enzyme. PCR-RFLP analysis revealed that this change was absent in 75 ethnically matched, unrelated normal controls. The proband was reportedly affected in the first decade of life, presented to us at 11 years of age, and was diagnosed with developmental lamellar cataract. His father aged 37 years at presentation was operated on for cataract in both eyes at age 30 years. The age of onset of the disease was not known in his case. The proband’s maternal uncle was normal upon ophthalmic evaluation.

**Figure 4:**
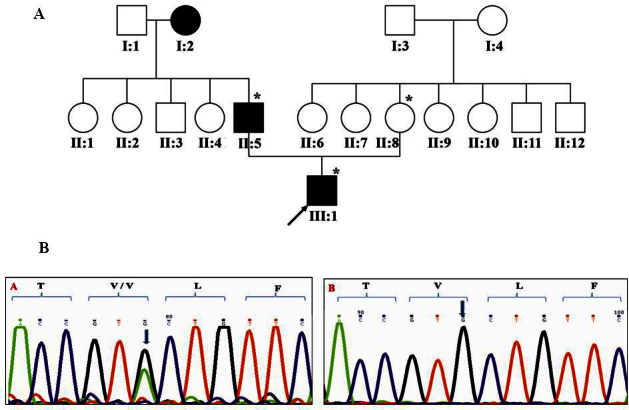
Family 39 with substitution c.84 G>A/Val28Val in *GJA3*. **A**: The pedigree of family 39 with autosomal dominant cataract is shown. Symbols marked with asterisks represent individuals who were genetically tested. **B**: Representative electropherograms of the individuals screened in family 39 show a silent mutation in *GJA3*. Heterozygous c.84G>A change was found in affected members (left panel) and a normal sequence in unaffected individuals (right panel). The arrow indicates sequence alteration.

In family 39 (Figure 4A), a heterozygous substitution of c.84G>A in *GJA3*/connexin 46 resulting in a silent mutation Val28Val was observed in affected individuals, i.e., the proband and his father (III:1 and II:5, respectively) whereas the proband’s mother (II:8) had two normal alleles (Figure 4B). The observed change is predicted to be in the first transmembrane domain of the *GJA3*/Cx46 protein [18] and was found to segregate with the disease phenotype in the three available family members. The change was absent in the normal control population screened with automated direct sequencing. The proband presented to our clinic at 1 year 4 months of age and was reported to be affected within the first year of life. He had undergone cataract surgery in the right eye and had posterior subcapsular cataract and nystagmus in the left eye. His father, aged 28 years at the time of examination, was reported to have been affected since early childhood and had undergone bilateral cataract surgery at age 5 years. The proband’s mother was unaffected except myopia in both eyes at age 28 years.

The potential impact of the missense changes identified in this study was assessed using bioinformatic tools, SIFT and PolyPhen2. Val196Met and Pro199Ser in *GJA8* and Pro197Ser in *GJA3* were predicted to have a “probably damaging” effect on protein function with a score of 1.0. The substitution of Val196Met in the *GJA8* gene was found to have a SIFT score of 0.06 and hence predicted as tolerated. Analysis of the silent mutation Val28Val in the *GJA3* gene for any splicing effects using the Human Splicing Finder, predicted a significant effect on the mRNA splicing compared with the wild-type sequence. This change was predicted to result in the creation of a new potential branch point, with a consensus value for the wild-type and the mutant sequence at 39.21 and 68.84, respectively. In addition, a putative splice enhancer motif (calculated based on the exonic splicing enhancer finder matrices for SRp40, SC35, SF2/ASF, and SRp55 proteins between the wild-type reference and the mutant sequence) was predicted to be broken with a score of −100.

## Discussion

Congenital hereditary cataracts are one of the leading causes of treatable blindness in children and are treated by surgical removal of the lens. Although more than 20 genes have been identified for non-syndromic autosomal dominant and recessive cataracts, there is possibly even more heterogeneity with more genes remaining to be identified [10].

The connexin (Cx) gene family encodes gap junction proteins that form gap junction channels that allow the passage of ions (K^+^, Ca^2+^) and small molecules (<1 kDa) including metabolites (e.g., glucose) and second messengers (inositol triphosphate [IP3], cyclic adenosine monophosphate, and cyclic guanosine monophosphate) between adjacent cells [19-21]. In non-chordate animals, a family of proteins called innexins forms these channels while in chordates connexin proteins form hexamers known as connexons in cell membranes. A gap junction channel contains 12 subunit proteins (connexins) distributed among two coaxially aligned hexameric hemichannels or connexons, located in the plasma membranes of the apposing cells. Channels can cluster at appositional membranes and form gap junctional plaques [22]. The human connexin genes are commonly divided into α, β, and an unnamed third group, all of which code for gap junction channels of different molecular weights [23]. At least 20 connexin genes have been identified in humans with proteins ranging from 25 to 62 kDa [24,25]. Mutations of specific connexin genes have been associated with several diseases including genetic deafness [26], skin disease [27], peripheral neuropathies [28], heart defects [29], and cataracts [30,31].

Of the 40 families screened in this study, we identified four potentially pathogenic sequence changes in four families. Including two families in which mutations in the *GJA8*/Cx50 and LIM2 genes were identified in our study earlier [13,14], mutations were found in the selected genes in six families. In the remaining cases, one or more mutations may have been present but not detected due to the sensitivity limit of the SSCP method, which is in the range of 70%–80%. Of these, two missense mutations (Val196Met and Pro199Ser) were in *GJA8*, and one missense (Pro197Ser) and a silent mutation (Val28Val) predicted to affect splicing were found in *GJA3* in separate families. Except for the Val196Met substitution in *GJA8*/Cx50 found in a family with autosomal recessive cataract, all the other mutations were associated with dominant cataracts. In family 32, the proband was homozygous for Val196Met and presented with total cataract at age 1.8 years while both parents and the other family members evaluated were heterozygous for the same and had normal lenses.

At least 27 mutations have been identified in different domains of Cx50 [11], in families with congenital cataracts to date, including the two described in the present study. Of the seven mutations reported in the Indian population, four, including Val196Met, Pro199Ser (this study), Thr203AsnfsX47 [14], and Arg198Gln [12], are located in the second extracellular loop of the connexin 46 protein, Val79Leu [32] and Pro88Gln [33] are in the second transmembrane domain, and Val44Glu [12] is in first transmembrane domain.

Cx46 is predominantly expressed in the lens and functions in gap junction communications between elongated fiber cells [34]. At least 25 mutations have been reported in the literature to date in GJA3/Cx46 in families with autosomal dominant congenital cataracts [11]. Of all mutations, five are reported from the Indian population. Two of these, Val28Val and Pro197Ser (this study), are located in the first transmembrane domain and the second extracellular loop of the connexin 50 protein, respectively. Of the other three, Val28Met [18] and Arg33Leu [35] are located in the first transmembrane domain, and Thr87Met [36] is in the second transmembrane domain.

The proband with the Val28Val silent change in *GJA3*/Cx46 presented to us within the first year of life and was pseudophakic in the right eye and had a posterior subcapsular cataract in the left with nystagmus. In comparison, a missense change of the same residue, Val28Met, was identified in an Indian family in which the proband had total cataract [18]. The impact of the isocoding change we identified is not clear, though it may possibly affect mRNA splicing. This change was predicted to result in the creation of a new potential branch point, and a putative splice enhancer motif for the linked SR protein (SC35) was predicted to be broken with a score of −100. There was no predicted effect of the observed change regarding a potential splice site, silencer, or any other motifs. These data suggest that mutations in connexin genes are a frequent cause of congenital hereditary cataract giving rise to dominant and recessive forms of disease.
